# AI-based diagnosis of depression and cardiovascular disease comorbidity based on big data

**DOI:** 10.1192/j.eurpsy.2025.383

**Published:** 2025-08-26

**Authors:** V. Pezoulas, K. Dobretz, G. Ehret, D. Fotiadis, A. Sakellarios

**Affiliations:** 1 Dept. of Materials Science and Engineering, University of Ioannina, Ioannina, Greece; 2 Department of Internal Medicine Hôpitaux, Universitaires de Genève, Genève, Switzerland; 3 Dept. of Mechanical and Aeronautics Engineering, University of Patras, Patras, Greece

## Abstract

**Introduction:**

There is strong clinical evidence that patients with depression have high probabilities to present a cardiovascular disease and vice versa. Thus, it is important to accurately identify these patients in order to provide the optimal management of the comorbid conditions.

**Objectives:**

To identify patients who have depression and cardiovascular disease using social and molecular biomarkers which are routinely collected in the clinical practice.

**Methods:**

Data from 502,379 participants in the UK Biobank were utilized in this work. A subset of the participants has a mental assessment using questionnaires about the presence of depression. CVD assessment was also available for the majority of the patients. In total, 126,033 participants had clinical assessment of both depression and CVD. From these, 8,925 patients had both comorbid conditions. An automated medical data curation tool described in a previous study was utilized to detect and mitigate data inconsistencies and elevate the input data integrity and usability. Hybrid boosting ensembles, including the XGBoost algorithm with a customized hybrid loss function was trained on the curated data, to reduce training and testing loss and to avoid overfitting effects. Dropout rates from deep learning theory were used in the hybrid loss function to further reduce biases during the decision-making process by controlling for the shape of the loss function. Random downsampling with replacement was also applied to match the control and target populations due to the increased class imbalance (ratio?) and with respect to the pre-defined set of confound factors. Additional classifiers including bagging classifiers were used for comparison purposes. The classification performance was assessed based on stratified 10-fold cross validation, where various metrics like the accuracy, sensitivity, specificity and area under the ROC curve scores were estimated. Advanced feature selection methods from coalition game theory, including the Shapley additive explanation (SHAP) exploratory analysis was utilized to identify predictors with positive or negative impact to have both the comorbid conditions These explanations were based on the classification outcomes from specific training and testing instances.

**Results:**

The XGBoost classifier had the best performance among all tested classifiers. The results were 0.85, 0.88, 0.81 and 0.92 for the accuracy, sensitivity, specificity and AUC, respectively. The figure 1, presents the explainability analysis for the selected biomarkers. As shown, there are simple social questions, but also some blood biomarkers which can be used for the identification of the patients with both the comorbid conditions of depression and CVD.

**Image 1:**

**Figure:**
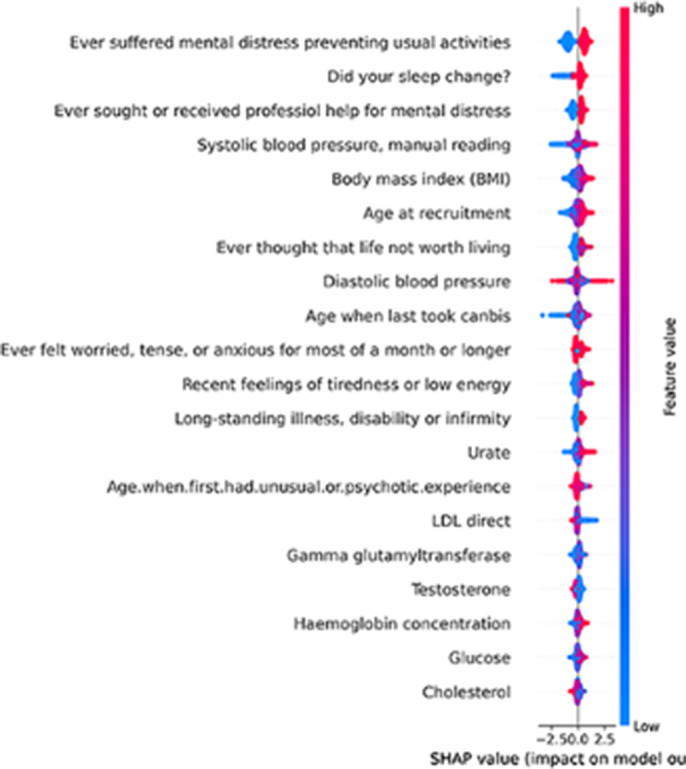

**Conclusions:**

We developed an AI-based approach which can diagnose depression and CVD to patients in a cost effective way with accuracy of 85% and AUC equal to 0.92.

**Disclosure of Interest:**

None Declared

